# Changes in muscle ultrasound for the diagnosis of intensive care unit acquired weakness in critically ill patients

**DOI:** 10.1038/s41598-021-97680-y

**Published:** 2021-09-14

**Authors:** Weiqing Zhang, Jun Wu, Qiuying Gu, Yanting Gu, Yujin Zhao, Xiaoying Ge, Xiaojing Sun, Jun Lian, Qian Zeng

**Affiliations:** Department of Critical Care Medicine, Rui Jin Hospital, Shanghai Jiao Tong University School of Medicine, No.197, Rui Jin Er Road, Shanghai, China

**Keywords:** Medical research, Signs and symptoms

## Abstract

To test diagnostic accuracy of changes in thickness (TH) and cross-sectional area (CSA) of muscle ultrasound for diagnosis of intensive care unit acquired weakness (ICU-AW). Fully conscious patients were subjected to muscle ultrasonography including measuring the changes in TH and CSA of biceps brachii (BB) muscle, vastus intermedius (VI) muscle, and rectus femoris (RF) muscles over time. 37 patients underwent muscle ultrasonography on admission day, day 4, day 7, and day 10 after ICU admission, Among them, 24 were found to have ICW-AW. Changes in muscle TH and CSA of RF muscle on the right side showed remarkably higher ROC-AUC and the range was from 0.734 to 0.888. Changes in the TH of VI muscle had fair ROC-AUC values which were 0.785 on the left side and 0.779 on the right side on the 10th day after ICU admission. Additionally, Sequential Organ Failure Assessment (SOFA), Acute Physiology, and Chronic Health Evaluation II (APACHE II) scores also showed good discriminative power on the day of admission (ROC-AUC 0.886 and 0.767, respectively). Ultrasonography of changes in muscles, especially in the TH of VI muscle on both sides and CSA of RF muscle on the right side, presented good diagnostic accuracy. However, SOFA and APACHE II scores are better options for early ICU-AW prediction due to their simplicity and time efficiency.

## Introduction

Loss of muscle mass is the clinical manifestation of critical illness neuromyopathy and usually involves bilateral symmetrical limb weakness^1^. Typically, it is defined as an intensive care unit acquired weakness (ICU-AW) and presents as flaccid quadriparesis with hyporeflexia or areflexia^2^. ICU-AW is a very strong indicator of disease severity and can result in a profound impact on outcomes, thus increasing the extent of mechanical ventilation, prolonging ICU length of stay and hospitalization, declining long-term functional status, and increasing death rate^3^. Farhan et al. reviewed the incidence of ICU-AW in 10 studies, with 25–31% incidence in medical ICU and 56–74% incidence in surgical ICU^4^. The incidence of ICU-AW in mechanically ventilated patients was about 50%, while in patients with systemic inflammatory response syndrome and severe sepsis, the incidence of ICU-AW can be as high as 70%, and even 100% when complicated by multiorgan dysfunction^2, 5, 6^. A study states that 1 year after ICU discharge, all patients who were diagnosed with acute respiratory distress syndrome complained of decreased physical function^7^. Patients who survived severe sepsis showed a functional disability and cognitive impairment that persisted for at least 8 years^8^. Aside from these physical and functional impacts, more than 50% of survivors suffered from mental disorders such as depression or anxiety^9^ which in turn led to much higher healthcare costs^10, 11^.

Nowadays, the diagnosis of ICU-AW can be qualified through strength assessment using six points Medical Research Council (MRC) score^12^, which is, in general, the accepted standard for diagnosis of ICU-AW^13^. The occurrence of ICU-AW is characterized by a mean MRC value under 4 per muscle group (12 muscle groups in total) or an MRC sum score under 48 for 12 muscle groups. However, strength assessment needs patients to be conscious, attentive, and able to comprehend simple verbal orders during testing. However, many critically ill patients will not be able to meet these prerequisites of strength assessment due to serious illness status, mechanical ventilation, and the use of anesthetic medications. Additionally, electrophysiological recordings or muscle biopsy are not routinely carried out in the majority of ICU. Consequently, exploration or development of alternative methods or technology for diagnosis of ICU-AW is direly needed for intensivists.

Muscle ultrasound is a convenient approach to investigate the muscle changes over time after admission in ICU^14^. Some muscle ultrasound studies have been able to detect reduction tendency of the cross-sectional area (CSA)^15, 16^ or decreasing pennation angle^17^, decrescent muscle thickness (TH)^15, 18^ and increase in echo intensity^17, 19, 20^ in patients who were critically ill. Nevertheless, the relation between those muscle parameters and ICU-AW remains unclear. Witteveen ‘s research tested the accuracy of neuromuscular ultrasound^21^ and found that receiver operating characteristics with a calculated area under the curve (ROC-AUC) of muscle parameters showed the promising possibility in differentiating patients with and without ICU-AW. The hypothesis of the present study is the changes in muscle ultrasound over time may show better diagnostic efficiency in the occurrence of ICU-AW. Consequently, we carried out the present study at a single center to test the diagnostic accuracy of the changes of muscle ultrasound over time in differentiating patients with and without ICU-AW.

## Methods

### Population and design

This longitudinal observational study was designed to be carried out at a single center, a general ICU in Shanghai, China from June 2019 to May 2020. The study was duly approved by the Rui Jin Hospital Ethics Committee and was performed in accordance with relevant guidelines and regulations. Written informed consent (either directly or through an appropriate surrogate) was obtained from all patients. Patients aged ≥ 18 years with an anticipated ICU stay of at least 2 days were eligible for screening after being evaluated daily for awakening and reaction to simple verbal commands. Exclusion criteria comprised individuals with prior diagnosed diseases characterized by generalized or regional weakness or with any diagnosis at the time of admission making patients abnormal muscle strength and unable to follow commands (e.g., cardiac arrest, stroke, spinal injury, traumatic brain injury, or intracerebral infection), or delirium or dementia during the ICU stay. Additionally, the patients experiencing edema of upper and lower limbs and patients who did not have arms or legs for muscle strength testing or ultrasound or had wounds, fractures, lesions, burns, or bleeding at the measurement points were excluded as well. Finally, patients who received early mobilization or physical therapy during the observation period were removed from the statistics.

### Clinical data collection

Baseline data were collected after ICU admission and included age, sex, Body Mass Index (BMI), hand dominance, admission diagnosis, Sequential Organ Failure Assessment (SOFA) score, Acute Physiology, and Chronic Health Evaluation II (APACHE II) score, risk factors for polyneuropathy or myopathy (restraints, surgery, nutritional supports, mechanical ventilation, glucose peak concentration, glucocorticoid, use of sedative and analgesic) and comorbidities (cardiac dysfunction, respiratory failure, liver dysfunction, acute kidney injury, hypertension, diabetes mellitus and Multiple Organ Dysfunction Syndrome (MODS).

### Ultrasound protocol

**Figure 1:**
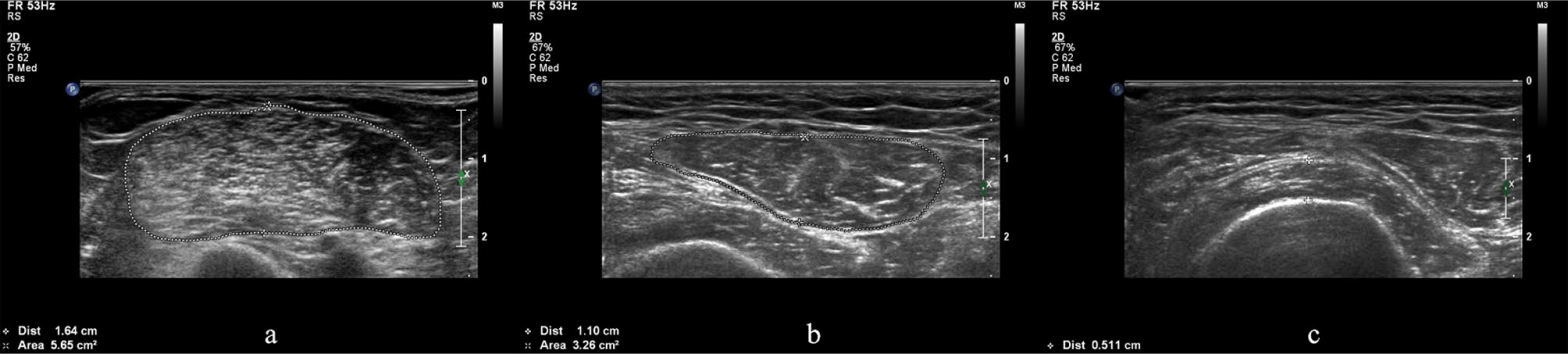
Ultrasound appearance of muscle thickness and cross-sectional area. Appearance and ultrasound measurement of thickness and cross-sectional area of biceps brachii muscle (**a**), rectus femoris muscle (**b**), and vastus intermedius muscle (**c**).

Two researchers who were trained and qualified to measure the muscle parameters immediately on the admission day by using a Philips ultrasound machine (IU22, USA) and a linear probe (frequency: 10–13 MHz) which enabled acquiring high-resolution images of clear structures of muscles^22^. Before performing, the patient must be in a supine position with extended elbows, wrists, knees and relaxed muscles, meanwhile the palms and toes of patients were facing or pointing to the ceiling^23^. The ultrasonography of muscles included TH and CSA of biceps brachii (BB) muscle, vastus intermedius (VI), and rectus femoris (RF) muscles (Fig. 1). All the muscles were measured bilaterally and scanned in the transversal (cross-sectional) image. The transducer was oriented transversally in relation to the longitudinal axis of the arm or thigh for obtaining a cross-sectional image, thus creating a right angle to the skin surface. Landmarks for ultrasound image acquisition were at standardized anatomical points, including the midpoint between supraglenoid tubercle and radial tuberosity for BB^24^, the second third of the distance between the anterior inferior iliac spine (AIIS), and the midpoint of the proximal border of the patella for RF, and the midline of the same distance as RF for VI^23^. The correlation coefficients of measurement accuracy of the two researchers were 0.88, 0.90, and 0.91 for BB muscle, RF muscle, and VI muscle respectively. When performing ultrasonography, the pressure on the skin was kept minimal, and adequate coupling agents were used for obtaining the images^25^. To enhance the accuracy of the measurement of target muscles, all the CSA and TH were measured three times continuously and an average was calculated as the final value. The whole muscle ultrasound procedure was repeated on day 4, day 7, and day 10 after ICU admission to know the changes of muscle TH and CSA.

### Muscle strength assessment

Another two researchers who were blind for the results of quantitative measurement of muscle parameters were in charge of assessing the conscious patients for muscle strength by using the MRC score on the 10th day after ICU admission^26^. The MRC score is extensively utilized for diagnosing the ICU-AW and its good interobserver reliability in critical settings has been confirmed in a previously published study^12^.For the patients mechanically ventilated with sedatives, if the RASS (Richmond Agitation Sedation Scale) fell anywhere between − 1 and 1^27^ and they showed a positive reaction to 5 verbal commands with facial muscles, we considered them feasible for muscle strength assessment^12^. Twelve muscle groups were tested for the calculation of MRC score including elbow flexion, wrist extension, shoulder abduction in upper limbs, and dorsiflexion of the foot, hip flexion knee extension in lower extremities. Examined subjects whose total MRC score < 48 were categorized as the ICU-AW group according to the international consensus statement^13^.

### Sample size

According to the equation of diagnostic experiment^28^, a significance level of 0.05 for a two-sided level and test power of 80% were assumed, and the expected sensitivity and specificity of muscle ultrasonography to be 0.8. Based on previous studies, ICU-AW was about 50% prevalent in critically ill patients^4^. 36 examined subjects was determined after considering the loss of 10% of the sample.

### Statistical analysis

Kolmogorov–Smirnov’s normality test was employed for evaluating continuous variables’ distribution. Data acquired from continuous variables with a normal distribution were expressed either as standard deviation or mean or as the interquartile range (IQR) or median in case if they had a non-normal distribution. Mann–Whitney test, Student t-test, exact Fisher test, and chi-squared test were employed to assess the differences among patients with and without ICU-AW diagnosis according to the distribution and type of the variable. Additionally, repeated measurement analysis of variance was used for testing the differences of changes in sonographic TH and CSA of observational muscles between groups.

The discriminative power of changes of muscle ultrasound over time was examined with a 95% confidence interval (CI) using ROC-AUC (receiver operating characteristic curves with calculated area under the curve). The discriminative power of AUC values among 90 and 100 percent have been described as < 60 percent as failed, 60–70 percent as poor, 70–80 percent as fair, and 80–90 percent as good^29^. The change of CSA and TH are represented by ΔCSA and ΔTH respectively, and was calculated using the formula: ΔCSA_day4/day7/day10_ = (CSA_day4/day7/day10_ − CSA_day1_)/CSA_day1_ or ΔTH_day4/day7/day10_ = (TH_day4/day7/day10_ − TH_day1_)/TH_day1_. Based on ROC curve analysis, the specificity, sensitivity, positive and negative predictive values (PPV, NPV) for muscle ΔCSA and ΔTH were calculated. The optimal cutoff value was confirmed by calculating the Youden Index. Youden Index = (specificity + sensitivity − 1). When the Youden Index is maximum, the corresponding value is the optimal cutoff value^30^. A significant two-level p-value taken for all analyses was < 0.05. SPSS version 19 was used for all statistical analyses.

## Results

**Figure 2:**
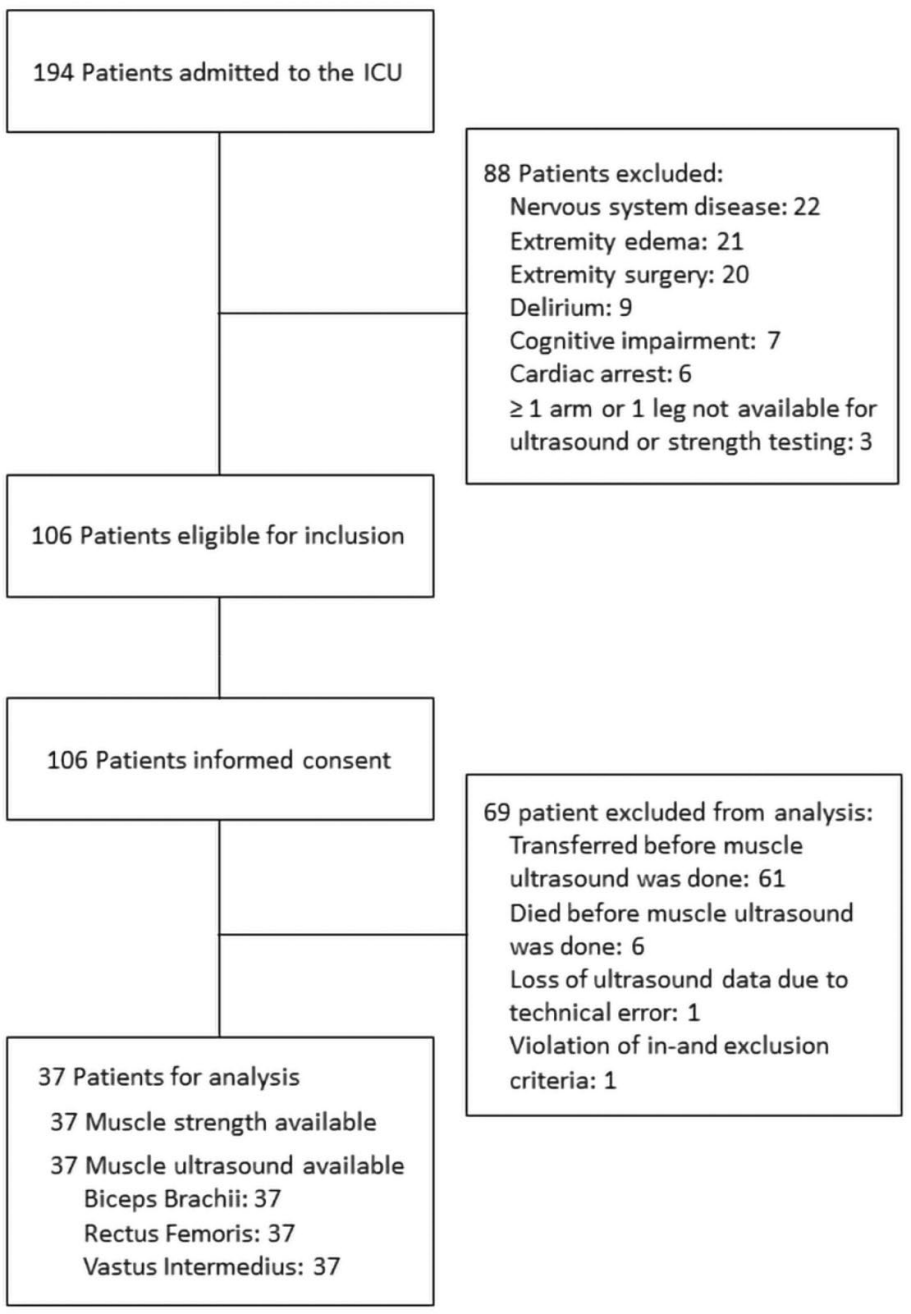
Flowchart representing screening and inclusion of patients.

In total, 106 patients were enrolled and their informed consent was obtained. Among them, 37 patients finally went through all 4 times muscle ultrasonography measurement successfully, of whom 24 had ICU-AW. The flowchart of screening and inclusion is shown in Fig. 2. Table 1 enlists the patient characteristics.

**Table 1:** Patient characteristics.

Variable	No ICU-AW n = 13	ICU-AW n = 24	t/χ^2^	P value
**Gender (%)**			0.794	0.373
Male	9(69.2)	13(54.2)		
Female	4(30.8)	11(45.8)		
Age, years (M ± SD)	48.92 ± 20.46	68.38 ± 15.11	− 3.297	0.002
MRC Score (M ± SD)	56.31 ± 5.22	31.29 ± 8.57	9.572	< 0.001
BMI (M ± SD)	22.85 ± 3.15	23.15 ± 3.56	− 0.250	0.804
**Diagnosis (%)**			16.207	0.001
Sepsis (%)	1(7.7)	11(45.8)		
Pneumonia (%)	1(7.7)	5(20.8)		
Severe pancreatitis (%)	8(61.5)	1(4.2)		
Others (%)	3(23.1)	7(29.2)		
**Surgery**			7.309	0.017
Yes (%)	6(46.2)	21(87.5)		
No (%)	7(53.8)	3(12.5)		
**Mechanical ventilation**			18.100	< 0.001
Yes (%)	4(30.8)	23(95.8)		
No (%)	9(69.2)	1(4.2)		
**Sedative (%)**			4.220	0.040
Yes (%)	3(23.1)	14(58.3)		
No (%)	10(76.9)	10(41.7)		
**Analgesic (%)**			13.017	0.001
Yes (%)	3(23.1)	20(83.3)		
No (%)	10(76.9)	4(26.7)		
APACHE II Score (M ± SD)	14.62 ± 6.32	22.21 ± 7.72	− 3.034	0.005
SOFA Score (M ± SD)	3.15 ± 3.00	9.00 ± 4.00	− 4.605	< 0.001
**Vasopressor (%)**			7.537	0.006
Yes (%)	2(15.4)	15(62.5)		
No (%)	11(84.6)	9(37.5)		
**Restraint (%)**			13.017	0.001
Yes (%)	3(23.1)	20(83.3)		
No (%)	10(76.9)	4(16.7)		
Peak Glucose, mmol/L (M ± SD)	11.69 ± 4.31	12.32 ± 3.73	− 0.469	0.642
**Glucocorticoid (%)**			1.768	0.538
Yes (%)	0(0)	3(12.5)		
No (%)	13(100)	21(87.5)		
**Nutritional support (%)**			1.377	0.440
Yes (%)	11(84.6)	16(66.7)		
No (%)	2(15.4)	8(33.3)		
**Hypertension**			5.247	0.022
Yes (%)	3(23.1)	15(62.5)		
No (%)	10(76.9)	9(37.5)		
**Diabetes (%)**			3.012	0.119
Yes (%)	1 (7.7)	8(33.3)		
No (%)	12(92.3)	16(66.7)		
**Cardiac dysfunction (%)**			2.295	0.216
Yes (%)	1(92.3)	7(29.2)		
No (%)	12(7.7)	17(70.8)		
**Respiratory failure (%)**			12.103	0.001
Yes (%)	5(38.5)	22(91.7)		
No (%)	8(61.5)	2(8.3)		
**Acute kidney injury (%)**			1.279	0.305
Yes (%)	3(23.1)	10(41.7)		
No (%)	10(76.9)	14(58.3)		
**Liver dysfunction (%)**			0.426	0.724
Yes (%)	4(30.8)	10(41.7)		
No (%)	9(69.2)	14(58.3)		
**MODS (%)**			8.479	0.003
Yes (%)	0(0)	11(45.8)		
No (%)	13(100)	13(54.2)		

ICU-AW, intensive care unit acquired weakness; MRC, Medical Research Council; BMI, Body Mass Index; APACHE II, acute physiology and chronic health evaluation II; SOFA, Sequential Organ Failure Assessment; M, mean; SD, standard deviation; IQR, interquartile range; MODS, multiple organ dysfunction syndrome.

**Figure 3:**
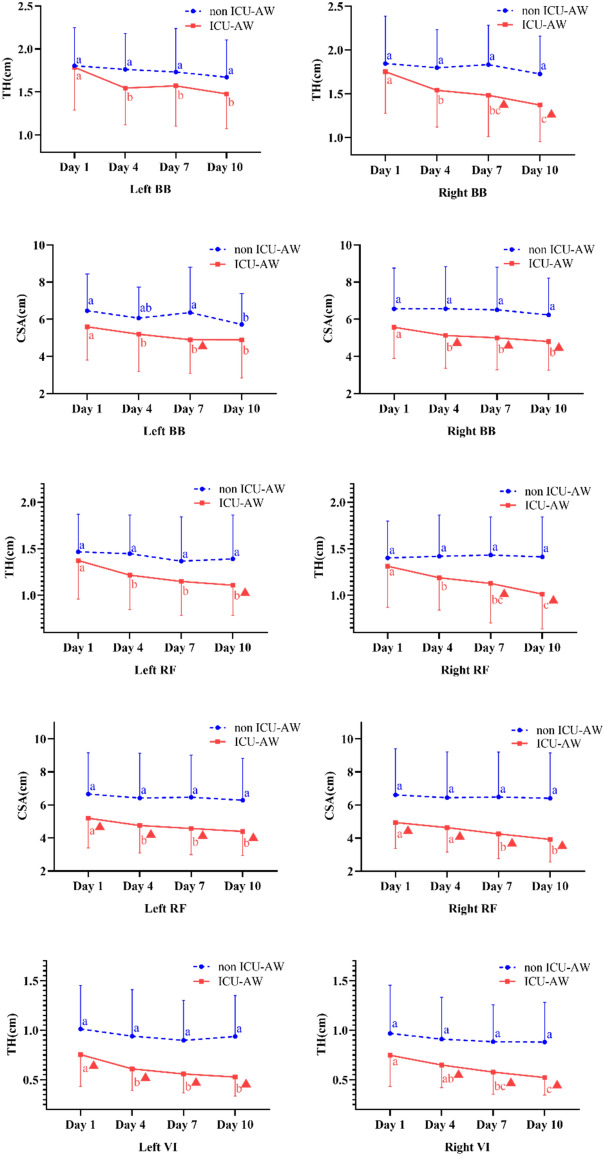
Differences in changes of muscles TH and CSA in patients with or without ICU-AW by repetitive measures analysis. BB, biceps brachii; RF, rectus femoris; VI, vastus intermedius; TH, thickness; CSA, cross-sectional area; a,b,c: different letters means significant differences within group, otherwise not ▲: significant differences between groups at the corresponding point in time.

Whether or not patients suffered from ICU-AW, all the groups presented a descending trend of both TH and CSA bilaterally. In the upper limbs, the changes of CSA in BB on the right side showed more statistical differences at different observation time points between groups. Moreover, ICU-AW patients had a greater degree of declination in CSA of RF bilaterally, and a remarkable reduction of TH in VI as well. Many significant differences between groups were found at different points in time (Fig. 3).

**Figure 4:**
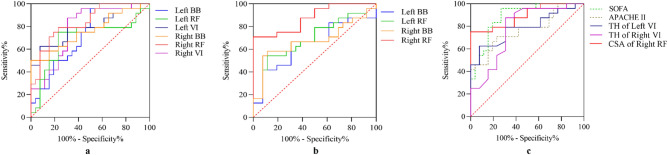
Comparison of ROC curves among SOFA score, APACH II score, ΔTH_day10,_ and ΔCSA_day10_ of muscles. (**a**) Comparison of changes in TH of biceps brachii muscle, vastus intermedius muscle and rectus femoris muscles; (**b**) comparison of changes in CSA of biceps brachii muscle, vastus intermedius muscle, and rectus femoris muscles; (**c**) comparison among changes in TH and CSA of muscles, SOFA and APACHE II score. BB, biceps brachii; RF, rectus femoris; VI, vastus intermedius; TH, thickness; CSA, cross-sectional area; APACHE II, Acute Physiology and Chronic Health Evaluation II; SOFA, Sequential Organ Failure Assessment.

The ΔTH of BB on both sides had higher ROC-AUC than ΔCSA of BB in the upper limbs, with ROC-AUC ranging from 0.702 to 0.792. The ROC-AUC of ΔCSA of BB on both sides were not significant except ΔCSA_day4_ of BB on the right side. In the lower limbs, most ROC-AUC of ΔTH and ΔCSA of RF were not significant on the left side. While the ROC-AUC of ΔTH and ΔCSA of RF on the right side were significantly higher, ranging from 0.734 to 0.888, especially ΔCSA_day10_ of RF (ROC-AUC: 0.888, p < 0.001). Besides, ΔTH_day10_ of VI had fair ROC-AUC values that were 0.785 on the left side and 0.779 on the right side (Table 2; Fig. 4).

**Table 2:** Receiver Operating Characteristic curves of changes in thickness and cross-sectional area of muscles over time.

Muscles	Lateral	Variable	ROC-AUC	SE	P	95% CI for ROC-AUC	
						Lower	Upper
BB	Left	**Thickness**					
		ΔTH_day4_	0.782	0.076	0.005	0.632	0.932
		ΔTH_day7_	0.705	0.087	0.042	0.535	0.875
		ΔTH_day10_	0.708	0.095	0.039	0.523	0.894
		**Cross-sectional area**					
		ΔCSA_day4_	0.551	0.094	0.611	0.367	0.735
		ΔCSA_day7_	0.676	0.090	0.080	0.500	0.853
		ΔCSA_day10_	0.647	0.092	0.143	0.466	0.828
	Right	**Thickness**					
		ΔTH_day4_	0.702	0.090	0.045	0.526	0.877
		ΔTH_day7_	0.792	0.081	0.004	0.632	0.951
		ΔTH_day10_	0.756	0.078	0.011	0.603	0.910
		**Cross-sectional area**					
		ΔCSA_day4_	0.728	0.083	0.024	0.566	0.889
		ΔCSA_day7_	0.692	0.086	0.056	0.524	0.861
		ΔCSA_day10_	0.676	0.090	0.080	0.500	0.852
RF	Left	**Thickness**					
		ΔTH_day4_	0.747	0.086	0.014	0.578	0.915
		ΔTH_day7_	0.583	0.094	0.408	0.399	0.768
		ΔTH_day10_	0.696	0.092	0.052	0.515	0.876
		**Cross-sectional area**					
		ΔCSA_day4_	0.593	0.093	0.356	0.410	0.776
		ΔCSA_day7_	0.689	0.088	0.061	0.516	0.862
		ΔCSA_day10_	0.699	0.088	0.049	0.527	0.870
	Right	**Thickness**					
		ΔTH_day4_	0.734	0.091	0.020	0.555	0.913
		ΔTH_day7_	0.779	0.080	0.006	0.622	0.936
		ΔTH_day10_	0.840	0.068	0.001	0.707	0.972
		**Cross-sectional area**					
		ΔCSA_day4_	0.635	0.092	0.181	0.454	0.815
		ΔCSA_day7_	0.843	0.068	0.001	0.710	0.976
		ΔCSA_day10_	0.888	0.052	< 0.001	0.785	0.990
VI	Left	**Thickness**					
		ΔTH_day4_	0.590	0.094	0.373	0.406	0.774
		ΔTH_day7_	0.660	0.088	0.112	0.487	0.833
		ΔTH_day10_	0.785	0.074	0.005	0.640	0.931
	Right	**Thickness**					
		ΔTH_day4_	0.571	0.106	0.484	0.364	0.777
		ΔTH_day7_	0.734	0.086	0.020	0.565	0.903
		ΔTH_day10_	0.779	0.086	0.006	0.611	0.947

BB, biceps brachii; RF, rectus femoris; VI, vastus intermedius; ΔTH, changes in thickness; ΔCSA, changes in cross-sectional area; SE, standard error; ROC-AUC, receiver operating characteristic curves with calculated area under the curve; CI, confidence interval.

Following that, we compared the diagnostic power of SOFA, APACHE II, and certain muscle parameters that showed good diagnostic performance as previously mentioned. The SOFA (ROC-AUC: 0.886, p < 0.001) and APACHE II scores (ROC-AUC: 0.767, p < 0.05) at the time of admission to the ICU showed close diagnostic efficacy compared to the changes in muscle parameters (Fig. 4).

Further, using specific thresholds (15% for ΔTH_day10_ of BB, RF and VI muscle, 12% for ΔCSA_day10_ of BB and RF) in term of Youden Index of ΔTH_day10_ and ΔCSA_day10_ that were calculated based on the ROC curve, the sensitivity, specificity, PPV, NPV, and accuracy were confirmed and are presented in Table 3. Diagnostic accuracy of ΔTH_day10_ and ΔCSA_day10_ of RF on the right side and ΔTH_day10_ of VI on both sides was high and ranged from 75.7 to 78.4%.

**Table 3:** Specificity, sensitivity, negative predictive value, positive predictive value and accuracy for defined cutoffs of ΔTH_day10_ and ΔCSA_day10_ of muscles.

Muscles	Lateral	Variable	Cutoff (%)	Sensitivity (%)	Specificity (%)	PPV (%)	NPV (%)	Accuracy (%)
BB	Left	ΔTH	15	36.5	76.9	80.0	45.5	59.5
		ΔCSA	12	66.7	46.2	66.7	38.5	56.8
	Right	ΔTH	15	58.3	76.9	83.3	52.6	67.6
		ΔCSA	12	58.3	84.6	82.4	50.0	64.9
RF	Left	ΔTH	15	58.3	76.9	83.3	52.6	67.6
		ΔCSA	12	58.3	76.9	50.0	47.4	62.2
	Right	ΔTH	15	70.8	84.6	89.5	61.1	75.7
		ΔCSA	12	70.8	100	70.8	84.6	75.7
VI	Left	ΔTH	15	79.2	69.2	82.6	64.3	75.7
	Right	ΔTH	15	83.3	69.2	83.3	69.2	78.4

BB, biceps brachii; RF, rectus femoris; VI, vastus intermedius; ΔTH, changes in thickness; ΔCSA, changes in cross-sectional area; PPV, positive predictive values; NPV, negative predictive values.

## Discussion

The present study confirmed that patients with ICU-AW had a significant reduction of muscle TH and CSA than those of patients without ICU-AW, especially in the lower extremities. Moreover, for a 15% threshold for ΔTH_day10_ and a 12% threshold for ΔCSA_day10_, muscles in lower extremities showed a good diagnostic accuracy of the diagnosis of ICU-AW, particularly on the right side. More importantly, changes in muscle ΔTH_day10_ and ΔCSA_day10_ of the lower extremities were found to have close diagnostic validity to SOFA and APACHE II scores at the time of ICU admission.

In this study, of all evaluated patients, 64.9% were found to have ICU-AW. Whether or not patients had ICU-AW, all patients presented the descending trend of both TH and CSA bilaterally. Moreover, patients with ICU-AW had an obviously greater degree of declination in CSA of RF bilaterally, and a remarkable reduction of TH in VI of both sides as well. Turton et al. carried out a study on 22 ICU patients who were mechanically ventilated and performed an ultrasonographic assessment of the flexor compartment of the elbow joint, the vastus lateral muscle, and the medial head of the gastrocnemius muscle on admission and 10 days later. The results showed that the loss of muscle mass mainly occurred in the lower extremities and there was no change in the size of the flexor compartment of the elbow joint. Such data helps to justify the argument regarding investigating the lower extremities further as peripheral muscles, in patients with critical illnesses have more chances to undergo early disuse atrophy^31^. In particular, a 3-week follow-up study employed ultrasonography to evaluate RF muscle in terms of the morphological changes and found severe muscle mass loss in CSA and muscle diameter experienced by all the ICU trauma patients. By day 20, approximately 45% of rectus femoris muscle mass was lost^32^. Consequently, in comparison to upper limbs, lower limbs muscles experienced earlier and greater atrophy. The potential reason was given in an earlier study that assessed rectus femoris CSA and protein/DNA ratio over time. The results showed that during the first week, virtually all cases decreased in muscle mass. Lower limb muscle atrophy is considered to be the result of net catabolism due to the decrease of muscle protein synthesis and the simultaneous increase of protein decomposition relative to protein synthesis^16^.

Few previous studies tested muscle ultrasonography for the diagnosis of ICU-AW or prediction of prognosis or similar symptoms. One study diagnosed skeletal muscle loss by measuring the CSA of RF using ultrasound and compared it with frailty to predict the prognosis of critically ill patients. The outcomes of the study clearly show that the prediction value of adverse discharge tendency by bedside ultrasound in the diagnosis of skeletal myopenia was consistent with frailty^33^. Moreover, a prospective observational study found that the larger the CSA in RF on the day of admission, the lower the occurrence of the muscle fiber necrosis and muscle waste of RF^34^. In addition, Greening et al. demonstrated that an independent risk factor for unscheduled readmission or death is a smaller quadriceps muscle size measured by ultrasound^35^. These studies showed the potential diagnostic possibility for ICU-AW diagnosis. Further, Witteveen’s study performed ultrasonographic TH of the tibialis anterior muscle, biceps brachii muscle, flexor carpi radialis muscle, and the rectus femoris muscle thus finding that for these muscles, the diagnostic accuracy of muscle TH was rather low with ROC-AUC ranging from^21^ 51.3 to 68.0%. Nevertheless, CSA which is considered as a crucial property for contraction and strength of muscle was not fully explored for its relation with ICU-AW^14^. According to results, the changes in quantitative muscle ultrasound had good performance when analyzed on MRC criteria for the diagnosis of ICU-AW and the best cutoff ratio of reduction in muscle parameters for diagnosing ICU-AW using ultrasound is more than 15% for ΔTH_day10_ and more than 12% for ΔCSA_day10_ in the lower extremity of the right side, which endorses the use of muscle ultrasound as a supplementary tool for ICU-AW diagnosis.

Although changes in some muscle parameters over 10 days presented good diagnostic efficacy, a comparison showed that SOFA and APACHE II scores at the time of ICU admission had a more adequate advantage. Given the time efficiency and implementation efficiency, the ROC-AUC of SOFA and APACHE II scores were shown to be near to the change in muscle parameters, making it appear unnecessary to predict the occurrence of ICU-AW by a 10-day muscle observation. Many predictors for the occurrence of ICU-AW have been confirmed, and the SOFA and APACHE II scores can be considered as indicators of multiple high-risk factors integrated together^36, 37^. However, from previous reports, SOFA and APACHE II scores alone did not present sufficient diagnostic efficacy^38, 39^, so further validation of these results is needed considering the limited sample size of the present study.

Some limitations of this study deserve the necessary commentary. First, due to the limited availability of biopsy or electroneuromyography in the ICU, we could not classify patients with ICU-AW into the three subcategories (critical illness neuromyopathy (CINM), critical illness myopathy (CIM), and critical illness polyneuropathy (CIP)). Second, we did not observe other ultrasonographic characteristics of muscle in recognition of ICU-AW, for instance, pennation angle and echo intensity which may have better diagnostic value. Third, it was impossible for ultrasound examiners to be completely blind to the MRC score because the absence or presence of spontaneous movements gave the impression of muscle strength. Therefore, to improve the accuracy of muscle measurement, all CSA and TH were measured three times in a row, and the average value is calculated as the final value to minimize the performer's deviation.

## Conclusion

Ultrasound measurement of muscles can be used as a tool to assist in the recognition of ICU-AW, especially for unconscious critically ill patients. Changes in TH and CSA of RF on the right side and the changes in TH of VI on both sides had good diagnostic accuracy for diagnosis of ICU-AW. However, considering the convenience and time efficiency, SOFA and APACHE II score are better options for early prediction of ICU-AW.

## References

[1] Kress JP, Hall JB (2014). ICU-acquired weakness and recovery from critical illness. N Engl J Med.

[2] Stevens RD (2007). Neuromuscular dysfunction acquired in critical illness: a systematic review. Intensive Care Med.

[3] Ali NA (2008). Acquired weakness, handgrip strength, and mortality in critically ill patients. Am J Respir Crit Care Med.

[4] Farhan H, Moreno-Duarte I, Latronico N, Zafonte R, Eikermann M (2016). Acquired muscle weakness in the surgical intensive care unit: nosology, epidemiology, diagnosis, and prevention. Anesthesiology.

[5] Connolly BA (2013). Clinical predictive value of manual muscle strength testing during critical illness: an observational cohort study. Crit Care.

[6] Kasotakis G (2012). The surgical intensive care unit optimal mobility score predicts mortality and length of stay. Crit Care Med.

[7] Herridge MS (2003). One-year outcomes in survivors of the acute respiratory distress syndrome. N Engl J Med.

[8] Iwashyna TJ, Ely EW, Smith DM, Langa KM (2010). Long-term cognitive impairment and functional disability among survivors of severe sepsis. JAMA.

[9] Lipshutz AK, Gropper MA (2016). Intensive care unit-acquired muscle weakness: an ounce of prevention is worth a pound of cure. Anesthesiology.

[10] Casaer MP (2015). Muscle weakness and nutrition therapy in ICU. Curr Opin Clin Nutr Metab Care.

[11] Hermans G (2014). Acute outcomes and 1-year mortality of intensive care unit-acquired weakness: a cohort study and propensity-matched analysis. Am J Respir Crit Care Med.

[12] De Jonghe B (2002). Paresis acquired in the intensive care unit: a prospective multicenter study. JAMA.

[13] Fan E (2014). An official American Thoracic Society Clinical Practice guideline: the diagnosis of intensive care unit-acquired weakness in adults. Am J Respir Crit Care Med.

[14] Bunnell A, Ney J, Gellhorn A, Hough CL (2015). Quantitative neuromuscular ultrasound in intensive care unit-acquired weakness: a systematic review. Muscle Nerve.

[15] Puthucheary ZA (2017). Rectus femoris cross-sectional area and muscle layer thickness: comparative markers of muscle wasting and weakness. Am J Respir Crit Care Med.

[16] Puthucheary ZA (2013). Acute skeletal muscle wasting in critical illness. JAMA.

[17] Parry SM (2015). Ultrasonography in the intensive care setting can be used to detect changes in the quality and quantity of muscle and is related to muscle strength and function. J Crit Care.

[18] Palakshappa JA (2018). Quantitative peripheral muscle ultrasound in sepsis: muscle area superior to thickness. J Crit Care.

[19] Cartwright MS (2013). Quantitative neuromuscular ultrasound in the intensive care unit. Muscle Nerve.

[20] Patejdl R (2019). Muscular ultrasound, syndecan-1 and procalcitonin serum levels to assess intensive care unit-acquired weakness. Can J Neurol Sci.

[21] Witteveen E (2017). Diagnostic accuracy of quantitative neuromuscular ultrasound for the diagnosis of intensive care unit-acquired weakness: a cross-sectional observational study. Ann Intensive Care.

[22] Mourtzakis M, Wischmeyer P (2014). Bedside ultrasound measurement of skeletal muscle. Curr Opin Clin Nutr Metab Care.

[23] Galindo Martin CA, Monares Zepeda E, Lescas Mendez OA (2017). Bedside ultrasound measurement of rectus femoris: a tutorial for the nutrition support clinician. J Nutr Metab.

[24] Grimm A (2013). Muscle ultrasound for early assessment of critical illness neuromyopathy in severe sepsis. Crit Care.

[25] Hermans G, Van den Berghe G (2015). Clinical review: intensive care unit acquired weakness. Crit Care.

[26] Stevens RD (2009). A framework for diagnosing and classifying intensive care unit-acquired weakness. Crit Care Med.

[27] Sessler CN (2002). The Richmond Agitation-Sedation Scale: validity and reliability in adult intensive care unit patients. Am J Respir Crit Care Med.

[28] Li J, Fine J (2004). On sample size for sensitivity and specificity in prospective diagnostic accuracy studies. Stat Med.

[29] Mjaset C (2020). Criteria for success after surgery for cervical radiculopathy-estimates for a substantial amount of improvement in core outcome measures. Spine J.

[30] Fluss R, Faraggi D, Reiser B (2005). Estimation of the Youden Index and its associated cutoff point. Biom J.

[31] Turton P, Hay R, Taylor J, McPhee J, Welters I (2016). Human limb skeletal muscle wasting and architectural remodeling during five to ten days intubation and ventilation in critical care: an observational study using ultrasound. BMC Anesthesiol.

[32] Annetta MG (2017). Ultrasound assessment of rectus femoris and anterior tibialis muscles in young trauma patients. Ann Intensive Care.

[33] Mueller N (2016). Can sarcopenia quantified by ultrasound of the rectus femoris muscle predict adverse outcome of surgical intensive care unit patients as well as frailty? A prospective, observational cohort study. Ann Surg.

[34] Puthucheary ZA (2015). Qualitative ultrasound in acute critical illness muscle wasting. Crit Care Med.

[35] Greening NJ (2015). Bedside assessment of quadriceps muscle by ultrasound after admission for acute exacerbations of chronic respiratory disease. Am J Respir Crit Care Med.

[36] Horn J, Hermans G (2017). Intensive care unit-acquired weakness. Handb Clin Neurol.

[37] de Jonghe B, Lacherade JC, Sharshar T, Outin H (2009). Intensive care unit-acquired weakness: risk factors and prevention. Crit Care Med.

[38] Wieske L (2014). Early prediction of intensive care unit-acquired weakness using easily available parameters: a prospective observational study. PLoS ONE.

[39] Witteveen E (2020). Early prediction of intensive care unit-acquired weakness: a multicenter external validation study. J Intensive Care Med.

